# The dual correlations between core beliefs changes and emotion regulation among disaster-affected residents: the moderating role of religiousness orientation

**DOI:** 10.3389/fpsyg.2026.1710083

**Published:** 2026-04-09

**Authors:** Qinqing He, Bai Zhang

**Affiliations:** 1 School of Social and Public Administration, Lingnan Normal University, Zhanjiang, Guangdong Province, China; 2 Department of Religion, School of Philosophy, Wuhan University, Wuhan, China

**Keywords:** core beliefs changes, disaster-affected residents, emotion regulation, post traumatic growth, post-disaster psychological recovery, religiousness orientation, social capital sense

## Abstract

Natural disasters not only cause material losses but also profoundly impact the psychological world of affected individuals, particularly their core belief systems. This study innovatively proposes the subjective perceptual concept of social capital sense, expanding the limitations of traditional objective measurements of social capital. Focusing on residents in the earthquake-stricken area of Shigatse, Tibet, China, on January 7, 2025, this research explores the dual relationships between core beliefs changes and emotion regulation. The study examines the relationships between core beliefs changes and post-traumatic growth, core beliefs changes and social capital sense, post-traumatic growth and emotion regulation, and social capital sense and emotion regulation, as well as the moderating role of individuals’ religiousness orientation. A two-stage questionnaire survey was conducted with 553 affected residents, and structural equation modeling was employed for analysis. The findings reveal that core beliefs changes are significantly positively correlated with post-traumatic growth, and post-traumatic growth is significantly positively correlated with emotion regulation. Core beliefs changes are significantly negatively correlated with social capital sense, while social capital sense is significantly positively correlated with emotion regulation. Religiousness orientation moderates both the relationship between beliefs changes and post traumatic growth and the relationship between core beliefs changes and social capital sense. Among individuals with high religious orientation, the positive relationship between core beliefs changes and post-traumatic growth is stronger, and the negative relationship with social capital sense is weaker. This study provides a theoretical basis for post-disaster psychological interventions, suggesting that differentiated strategies based on the psychological dimensions of affected residents can promote more effective psychological recovery.

## 1 Introduction

Natural disasters represent severe challenges faced by human society. Their destructiveness is not only reflected in material losses but also profoundly impacts the psychological world of affected populations. As profoundly disruptive experiences, major disasters can drastically shake individuals’ core beliefs about themselves, others, and the world, forcing individuals into a period of cognitive and emotional reconstruction (Kannis-Dymand et al., 2019). During this process, the psychological responses of disaster victims are not limited to post traumatic stress alone; they may also contain opportunities for growth and transformation—that is, the positive psychological changes individuals experience after struggling with traumatic events (Craig et al., 2024).

Social capital sense, as individuals’ subjective evaluation and perception of the trust, norms, support, and resource availability embedded in their social networks, may undergo dynamic changes under specific circumstances (Chilenski et al., 2023). Following major natural disasters, existing social structures and interpersonal trust are severely disrupted, potentially leading to significant declines in disaster-affected residents’ assessment and perception of social capital.

Core beliefs changes serve as both an important prerequisite for the occurrence of post traumatic growth and may lead to decreased perceptions of social connections and support (i.e., social capital sense), thereby exerting complex and contradictory effects on emotion regulation abilities.

Furthermore, against the backdrop of diverse cultural and religious contexts, individuals’ religiousness orientation may play a significant moderating role in this complex psychological process. For individuals with high religiousness orientation, their belief systems may provide a transcendent framework for interpreting disasters, strengthen the positive transformation of core beliefs changes into post-traumatic growth, and buffer its negative correlation with social capital sense through facilitating meaning-making, providing community support, and promoting reflection (Van Tongeren et al., 2020). However, this potential moderating role has not been sufficiently empirically examined in disaster psychology research, particularly within China’s culturally diverse religious context.

On January 7, 2025, a strong earthquake struck Shigatse City in Tibet, with a maximum magnitude of 6.8, causing severe damage to multiple areas of the city. This study focuses on the affected residents of this city to explore the relationships among core beliefs changes, post traumatic growth, social capital sense, emotion regulation, and religiousness orientation in a post-disaster environment.

The significance of this study lies in its innovative introduction of the concept of “social capital sense,” which breaks through the limitation of traditional social capital research that primarily focuses on objective social structures and the quantity of social networks, shifting the research perspective toward individuals’ subjective perception and dynamic evaluation of social capital. This conceptual innovation is particularly applicable to post-disaster environments. In addition, it theoretically helps to clarify the multiple relationships between core belief changes and post-disaster psychological adaptation, breaking through the limitations of previous studies that only focused on a single correlational path. Practically, it can provide more precise guidance for post-disaster psychological aid, namely, adopting differentiated intervention strategies for affected residents with different religious backgrounds to promote their post traumatic growth and comprehensively enhance the effectiveness of post-disaster psychological reconstruction.

## 2 Literature review and hypotheses

### 2.1 Core beliefs changes

Core beliefs refer to individuals’ fundamental, absolute, and enduring beliefs about themselves, others, and the world (Beck and Dozois, 2011). According to schema theory, schemas are organized cognitive structures through which individuals understand the world, with core beliefs serving as central components of these schemas that influence how individuals process and interpret information (Knapp and Beck, 2008). Beliefs may change in response to factors such as personal success, patterns of social interaction, and institutional environments (Delhey and Newton, 2003).

The formation of core beliefs is closely related to individuals’ early life experiences, family environment, and social interactions. Particularly, negative experiences during childhood, such as emotional neglect, abuse, or overprotection, may lead individuals to develop negative core beliefs (Duru and Balkıs, 2022).

Traumatic events challenge individuals’ assumptive worlds, forcing them to reevaluate and adjust these assumptions. The change process involves cognitive processing of traumatic events, including self-blame, denial, and rumination, in order to adapt to new realities. These changes may lead to emotional responses such as anxiety, confusion, and helplessness, but they also represent important prerequisites for cognitive growth and adaptation. Successful cognitive processing may lead to positive changes in the assumptive world, enhancing individuals’ adaptive capacity and psychological resilience (Janoff-Bulman, 1989).

Deprivation of core needs (such as acceptance and sense of security) may lead to the destabilization of core beliefs (Clark et al., 1990). Factors such as gender, age, time since the traumatic event, reflective rumination, and depressive symptoms may influence the direction and extent of core beliefs changes (Romeo et al., 2022). When individuals experience negative life events, their core beliefs may be shaken, thereby triggering depressive symptoms. This process involves the activation of automatic thoughts, cognitive distortions, and negative cognitive biases (Beck, 2008). The process of Core Beliefs Change involves questioning and reevaluating original beliefs, representing an important step in individuals’ adaptation to new environments and cognitive reconstruction (Padesky, 1994). Research findings by Matsudaira et al. (2021) indicate that social changes and lifestyle alterations resulting from the pandemic significantly shook some individuals’ core beliefs, particularly those concerning sense of security and interpersonal relationships. Positive core beliefs changes may enhance individuals’ psychological resilience, promoting their adaptation and growth during the pandemic.

Core beliefs include positive or negative views of the world, as well as beliefs about justice and controllability. Changes in core beliefs may lead to either post traumatic stress disorder or post traumatic growth, depending on how individuals cope with and process these changes (Otani et al., 2017). Positive core beliefs changes help enhance psychological resilience, promote positive emotions, improve interpersonal relationships, and prevent depression and anxiety. These effects are typically accompanied by shifts from self-denial to self-acceptance, from pessimism to optimism, and from isolation to connection (Overholser et al., 2010).

### 2.2 Core beliefs changes and post traumatic growth

Major events, as sources of impact, may destabilize individuals’ original cognitive balance and trigger cognitive reconstruction (LoSavio et al., 2011).

Through meaning-making and positive coping strategies, individuals can achieve growth from major events, and this growth may be accompanied by positive adjustments in core beliefs (Tedeschi and Calhoun, 2004).

Post traumatic growth is typically accompanied by changes in the following areas: new possibilities, relating to others, personal strength, spiritual change, and appreciation of life (Tedeschi and Calhoun, 1996). Haspolat and Çırakoğlu’s (2021) study on individuals with post-traumatic experiences indicated that core beliefs disruption is a significant predictor of post traumatic growth.

Following major stressful events, individuals may reevaluate and potentially disrupt their original core beliefs. This disruption provides individuals with opportunities to reconstruct and adapt to new realities, serving as a prerequisite for post traumatic growth (Dominick et al., 2022).

Dominick (2022) examined individuals’ post traumatic growth, core beliefs disruption, and changes in social support during the COVID-19 pandemic. Results showed that over time, some individuals’ core beliefs gradually adapted to the challenges brought by the pandemic, achieving positive changes that were closely related to post traumatic growth and social support.

Traumatic or stressful events may challenge individuals’ life narratives- the story frameworks through which individuals understand their life experiences. These narratives depend on individuals’ core beliefs (Calhoun et al., 2000). Natural disasters may destroy the positive assumptions individuals held prior to the disaster, leading to negative world assumptions and stress responses. However, if individuals can reconstruct assumptions through cognitive adjustment, they may achieve post traumatic growth. Natural disasters themselves do not directly promote growth, but they can challenge individuals’ original core belief systems, trigger cognitive rumination, and thereby facilitate positive reconstruction of views about self, others, and the world. Based on the above theories, we propose:

*Hypothesis 1*: post-disaster core beliefs changes are positively associated with disaster-affected residents' post traumatic growth.

### 2.3 Core beliefs changes and social capital sense

An individual’s evaluation of their social capital depends not only on the objective social environment but also on their perception of the social environment they inhabit and the social capital they possess. Major life events (such as natural disasters, psychological trauma, career success) and changes in values may alter individuals’ assessment of the social capital they perceive as useful. Therefore, this study proposes the concept of social capital sense.

Blumberg et al. (2016) found that some adolescents may overestimate the value of virtual social capital, such as believing that having many followers represents high social status, thereby developing an inaccurate social capital sense.

The core components of social capital include trust, social network, and participation. The absence or reduction of these three dimensions directly weakens individuals’ perception of social capital (Guillen et al., 2011). Individuals’ subjective evaluation of social capital involves perceptions of social networks and participation, as well as feelings of trust and social support (Ahn and Davis, 2020).

If the quality of connections within social networks is low (e.g., primarily weak ties lacking deep interaction) or diversity is insufficient (e.g., overly narrow social circles), individuals’ ability to obtain resources and support from social networks may be limited. The absence of shared goals and activities among community members reduces opportunities for interaction and cooperation, thereby affecting sense of social belonging (Chang and Zhu, 2012).

Social identity is an important component of social capital. When individuals perceive value incongruence between themselves and others in their social networks or communities, their sense of social identity may be weakened (Al-Ghaith, 2015).

Previous research has demonstrated relationships between beliefs, values, and social capital sense.

Individuals with positive values are better able to cope with life challenges, reduce the impact of negative emotions, and thus maintain positive social interactions (Lakey and Cassady, 1990). Optimistic individuals are more likely to perceive the positive aspects of society and actively participate in social activities, thereby accumulating more social capital (Bezrukova et al., 2016).

The formation and maintenance of social capital may depend on shared core beliefs and values among members. Research by Stolle and Rochon (1998) found that increased member diversity may challenge the original shared beliefs and values within organizations, thereby indirectly affecting the formation of social capital among organizational members.

Social capital is closely related to interpersonal core beliefs (trust, reciprocity, and shared norms). As society changes (such as changes in community structure, family structure, and work nature), trust and reciprocal relationships between people are affected. Although the rise of the internet and social media has provided new ways of socializing, it has also weakened trust and reciprocity in traditional face-to-face interactions, further affecting people’s perception of social capital (Putnam, 2000).

Control beliefs are related to social capital; the absence or reduction of control beliefs affects individuals’ perception and utilization of support systems (Lefcourt et al., 1984).

Research by Imhoff and Bruder (2014) found that individuals with high conspiracy mentality are more likely to distrust authority institutions such as government and media, thereby weakening the “institutional trust” dimension of social capital. Individuals with strong conspiracy mentality exhibit widespread distrust of others and institutions, with relatively low social participation and willingness to cooperate. If individuals believe in conspiracy theories, they are more likely to perceive injustice and hidden control in society, thereby reducing positive evaluations of social capital (such as “society is fair” and “others are trustworthy”).

Research by Morgan et al. (2023) found that individuals who trust public health information are more likely to perceive community support and cooperation. Individuals with high levels of trust are more likely to participate in community activities, promoting information sharing and mutual assistance behaviors.

In previous research, social capital has been defined as an objective social environment. In disaster contexts, researchers have extensively focused on its impact on the psychology of affected residents (Wind et al., 2011; Delilah Roque et al., 2020; Uekusa et al., 2022). However, individuals’ evaluation and feelings about the social capital they possess, as well as the dynamic changes that may occur in natural disaster environments, have been overlooked.

In disaster environments, negative discrepancies between individuals’ initial expectations of social capital and actual feedback may directly lead to reduced levels of perceived social capital. In natural disaster environments, changes in individuals’ core beliefs may be accompanied by changes in traditional values or the emergence of alternative values, thereby triggering conflicts between personal and others’ values, consequently reducing individuals’ sense of social belonging and social capital sense. When individuals’ positive core beliefs (such as “the world is safe”) are shaken by disasters, their sense of trust in others (a key component of social capital) may decline sharply. An individual who perceives the world as dangerous and others as unpredictable can hardly perceive high levels of social capital. Furthermore, core beliefs changes may lead to social withdrawal and behavioral avoidance. Individuals may no longer be willing to participate in social activities or maintain social relationships, thereby cutting off the sources of social capital formation. Additionally, if individuals observe unequal resource distribution or bias in rescue efforts following disasters, they may question social justice. This questioning may weaken individuals’ trust and support for social institutions, thereby affecting social capital sense.

Based on the above theories, we propose:

*Hypothesis 2*: post-disaster core beliefs changes are negatively associated with disaster-affected residents' social capital sense.

### 2.4 Post traumatic growth and emotion regulation

Post traumatic growth involves the in-depth processing and expression of trauma-related emotions. It helps individuals express and release trauma-related emotions, reducing emotional suppression and accumulation through emotional release and reflection, thereby improving emotional states. Post traumatic growth prompts individuals to adopt more adaptive coping strategies, such as positively reinterpreting traumatic events and seeking social support, which help individuals better cope with stress and challenges and reduce psychological distress (Groleau et al., 2013).

Post traumatic growth can promote individuals’ seeking and utilization of social support, enhancing the effectiveness of social support networks. This social support not only provides emotional comfort but also facilitates mutual assistance and cooperation among individuals, helping them better cope with psychological challenges (Calhoun et al., 2010).

Post traumatic growth can improve interpersonal relationships, making individuals more adept at establishing and maintaining positive social connections. These relationships provide individuals with additional psychological resources, helping them maintain psychological balance when facing stress (Prati and Pietrantoni, 2009).

Post traumatic growth can enhance individuals’ psychological adaptability, enabling them to adapt more quickly to environmental changes and challenges (Zhou et al., 2017).

Post traumatic growth is typically accompanied by increases in positive emotions such as hope, optimism, and satisfaction, which help individuals more effectively regulate negative emotions such as anxiety and depression (Tedeschi and Calhoun, 1996).

A positive correlation exists between post traumatic growth and psychological resilience (Tedeschi et al., 2007). Individuals who experience post traumatic growth often demonstrate higher psychological resilience and are better able to cope with adversity and challenges. This psychological resilience not only helps individuals recover after trauma but also promotes their long-term mental health (Seligowski et al., 2015).

Rahimzadegan et al. (2022) demonstrated that post traumatic growth significantly and positively predicts the use of positive emotion regulation strategies, such as emotional expression and cognitive reappraisal. These strategies help patients more effectively process trauma-related negative emotions, reduce internal pressure through emotional expression, and decrease emotional distress by reappraising situations.

The cognitive reconstruction occurring during post traumatic growth enables individuals to view traumatic events in more flexible and open ways. This cognitive flexibility helps individuals adopt more effective coping strategies when facing stress. Post traumatic growth is typically negatively correlated with negative emotion regulation strategies, such as emotional suppression and avoidance (Larsen and Berenbaum, 2015).

During the process of post traumatic growth, individuals can learn to regulate their emotions more effectively. This enhancement of emotion regulation ability manifests in multiple aspects, including identifying and understanding one’s emotions, adopting appropriate strategies to regulate emotional intensity, and maintaining emotional stability. Individuals with high levels of post traumatic growth demonstrate greater diversity in emotion regulation strategies. They are not only able to select appropriate emotion regulation strategies based on situations but also can flexibly switch between different strategies (Wild and Paivio, 2004).

According to the Broaden-and-Build Theory of Positive Emotions, positive emotions function differently from negative emotions. Negative emotions narrow thought-action repertoires to address immediate threats, whereas positive emotions broaden individuals’ thought-action repertoires (e.g., inspiring exploration, integration behaviors) and build enduring personal psychological resources (Fredrickson, 2004). The experience of post traumatic growth (such as gratitude, discovering new possibilities, enhanced sense of personal strength) itself generates a series of positive emotions. Common negative emotions after natural disasters (such as fear, sadness) narrow cognition and maintain distress. The positive emotions stimulated by post traumatic growth can counteract or interrupt this negative cycle. The positive emotions brought by post traumatic growth build and strengthen the psychological resource pool (such as resilience, creativity, social connections) for coping with future stress. An individual who feels “more empowered” naturally regulates better when facing adversity. The capacity to accommodate both positive and negative emotions simultaneously is a hallmark of mental health. Post traumatic growth may not eliminate pain, but it allows positive experiences to coexist with pain, thereby enhancing overall emotion regulation ability.

In previous research, scholars have paid more attention to post traumatic growth because it holds greater long-term value for individuals experiencing trauma. Emotion regulation has often been defined as an explanatory variable, and its positive impact on or moderating role in post traumatic growth has been extensively discussed (Thomas et al., 2020; Kim et al., 2023; Al Ziadi et al., 2023). In this study, the relationship between post traumatic growth and emotion regulation among post-disaster residents was explored, proposing:

*Hypothesis 3*: post traumatic growth is positively correlated with emotion regulation among post-disaster residents.

### 2.5 Social capital sense and emotion regulation

Social capital encompasses individuals’ subjective perceptions of potential emotional support and resource availability within social interactions.

Social support networks play an important role in individuals’ emotion regulation, particularly for adolescents with weaker emotion regulation abilities (Zimmermann and Iwanski, 2014).

Supportive communication is one of the important pathways for emotion regulation. Individuals can effectively regulate their emotional states by sharing emotions with others and seeking advice or comfort. This process depends on individuals’ perception and utilization of social capital (Nozaki and Gross, 2025).

In Asian cultures, a significant correlation exists between interpersonal relationships and emotional suppression. Individuals often suppress emotions to maintain interpersonal harmony, and those with strong social capital are more likely to adopt suppression-based emotion regulation strategies (Butler and Gross, 2009).

Individuals’ subjective evaluation of social capital includes not only quantity (such as number of supporters) but also quality (such as trustworthiness of support). Research shows that subjectively perceived support quality (such as “feeling understood”) predicts emotion regulation outcomes better than objective quantity (Lopez et al., 2024).

If individuals lack clear awareness of their emotions, they may underestimate the value of social support and reduce help-seeking behaviors, thereby affecting their flexibility in emotion regulation (Singh and Young, 2020).

Social capital sense indirectly regulates emotions by influencing individuals’ social cognitive abilities (such as empathy and perspective-taking). Individuals with high Social capital sense may be better at understanding problems from others’ perspectives, thereby reducing egocentric emotional responses (Shuman, 2013).

Humans have a fundamental, universal need to establish and maintain lasting, positive, and stable emotional connections with others (i.e., sense of belonging). Satisfying this need is crucial for physical and mental health. The fulfillment of belongingness itself brings feelings of well-being and meaning in life, which form the foundation of good emotion regulation. In natural disaster environments, when individuals feel they are part of a whole, it can alleviate anxiety about loneliness, death, and meaninglessness of life. The care, concern, respect, or assistance derived from social networks are key resources for coping with stress and maintaining physical and mental health. Social capital sense is essentially individuals’ subjective perception and belief regarding the quantity and reliability of social support resources available to them. It is not only an evaluation of past support but also an expectation of whether future support will be available. Regardless of whether individuals are under stress, high levels of social capital sense can directly bring positive emotional experiences.

Based on the above theories, we propose:

*Hypothesis 4*: social capital sense is positively correlated with emotion regulation among post-disaster residents

### 2.6 Religiousness orientation, core beliefs changes, and post traumatic growth

The context of this study is Shigatse City, China, which experienced an earthquake on January 7, 2025. The research sample includes both religious and non-religious residents of this city. Therefore, the concept of religiousness orientation is employed. Through relevant quantitative work, a more comprehensive exploration of the psychological states and dynamic changes of residents in this city after the disaster can be achieved.

Religion may function as a cognitive schema that buffers the impact of external events on internal beliefs (Zukerman and Korn, 2014). It significantly influences post-traumatic growth through meaning-making processes (Gall et al., 2009).

Prieto-Ursúa and Jódar (2020) pointed out that religiousness indirectly promotes post traumatic growth by enhancing the sense of meaning in life, while spirituality directly affects personal growth.

Religiousness, as part of cultural worldviews, can buffer the negative post-disaster traumatic impacts. The emotional and practical support provided by religious groups can facilitate individuals’ positive reconstruction of core beliefs (Laufer et al., 2010).

Religious practices (such as meditation, prayer) may promote individuals’ transition from intrusive rumination to reflective rumination, thereby more positively integrating traumatic experiences (Lee and Kim, 2021).

For religious believers, ruminative cognitive processing is a key mechanism in the formation of their post traumatic growth (Calhoun et al., 2000).

Highly religious individuals may reconstruct the meaning of trauma through religious narratives, thereby reducing the transformation of negative core beliefs and making core beliefs changes more easily transformed into post traumatic growth (Shaw et al., 2005).

Religious beliefs may promote post traumatic growth by enhancing “sense of meaning.” For example, highly religious nurses may reinterpret stressful events as “sacred missions” or “spiritual tests” (Nowicki et al., 2024).

Positive religious coping is significantly positively correlated with post traumatic growth. Positive religious coping can enhance the impact on post traumatic growth through meaning reconstruction (such as “disasters are opportunities for spiritual growth”) (García et al., 2014).

Religious beliefs provide individuals with a framework that transcends daily difficulties, enabling them to find hope and purpose amidst the destructive impacts of natural disasters. Disaster-affected residents with high religiousness orientation are more likely to rely on religious beliefs as psychological buffers and resources when facing natural disasters, thereby reducing the psychological impact of disasters. The sense of meaning and purpose provided by religious beliefs helps individuals reevaluate and adjust their core beliefs to cope with disasters in more positive ways. Religious practices (such as meditation, prayer) encourage individuals to engage in reflective rumination rather than being troubled by intrusive rumination. This reflective rumination helps individuals more positively integrate traumatic experiences and reconstruct their worldviews and life perspectives, thereby promoting the emergence of post traumatic growth. Furthermore, religious narratives provide individuals with a framework for interpreting and coping with natural disasters. Through religious narratives, individuals can view traumatic experiences as tests or opportunities for growth, thereby reducing the transformation of negative core beliefs and facilitating the transformation of core beliefs changes into post traumatic growth. Disaster-affected residents with high religiousness orientation are more likely to adopt positive religious coping strategies, such as viewing disasters as opportunities for spiritual growth. This coping strategy can enhance individuals’ perception and experience of post traumatic growth, enabling them to draw strength from disasters and achieve personal growth. Therefore, we propose:

*Hypothesis 5*: religiousness orientation moderates the relationship between core beliefs changes and post traumatic growth. For individuals with high religiousness orientation, the positive correlation between core beliefs changes and post traumatic growth is stronger.

### 2.7 Religiousness orientation, core beliefs changes, and social capital sense

Previous research has explored the relationship between religion and social capital sense.

Kaasa’s (2013) research results indicated a significant positive correlation between religious beliefs and both cognitive social capital and structural social capital. Yeung (2004) found that church volunteers were more actively engaged in community connections and altruistic behaviors. Park and Bowman (2015) pointed out that religiosity and religious worldview identification significantly influence cross-racial interactions. Gemar (2024) indicated that religious identity significantly affects social network variety through multiple religious identities. Welch et al. (2004) demonstrated that both frequency of religious participation and religious guidance are significantly positively correlated with community trust, with the former operating through strengthening community belonging and the latter through providing a sense of meaning. Strømsnes (2008) noted that weak religious intensity (such as merely joining religious organizations without participating in activities) is insufficient to promote social capital, and that religious groups can strengthen social connections through regular collective activities (such as worship services, prayers).

Furthermore, religious practices (such as prayer, rituals) enhance trust among co-religionists through emotional resonance and collective memory (Hopkins, 2011). Religious participation (membership in a religious congregation) significantly promotes individuals’ connections with high-status groups (such as political officials, corporate executives, scientists, the wealthy) (Wuthnow, 2002). Religious rituals and collective activities strengthen bonding social capital through reinforcing internal cohesion (such as mutual assistance among co-religionists), while religious organizations promote bridging social capital through cross-group services (such as charitable activities) (Shapiro and Sharony, 2018).

Religion may influence social capital through organizational pathways (such as church volunteer activities) and individual pathways (such as private prayer), with the former enhancing structural social capital (such as formal networks) and the latter enhancing cognitive social capital (such as trust).

In natural disaster environments, individuals with religiousness orientation may reconstruct the meaning of disasters through religious narratives, thereby weakening the negative impact of core beliefs changes on post-disaster social capital sense. Additionally, in post-disaster contexts, the multiple social identities of religious individuals may bring more channels of social support, buffering the impact of core beliefs changes. Individuals with high religiousness orientation can accumulate social capital reserves through religious participation, and these reserves can buffer the negative impact of core beliefs disruption on post-disaster social capital sense in disaster environments. After disasters, religious individuals, especially those with high levels of religious participation, can obtain material and information assistance through church activities, maintaining higher levels of post-disaster social capital sense. Therefore, we propose:

*Hypothesis 6*: religiousness orientation moderates the relationship between core beliefs changes and social capital sense. For individuals with high religiousness orientation, the negative correlation between core beliefs changes and social capital sense is weaker.

**Figure 1:**
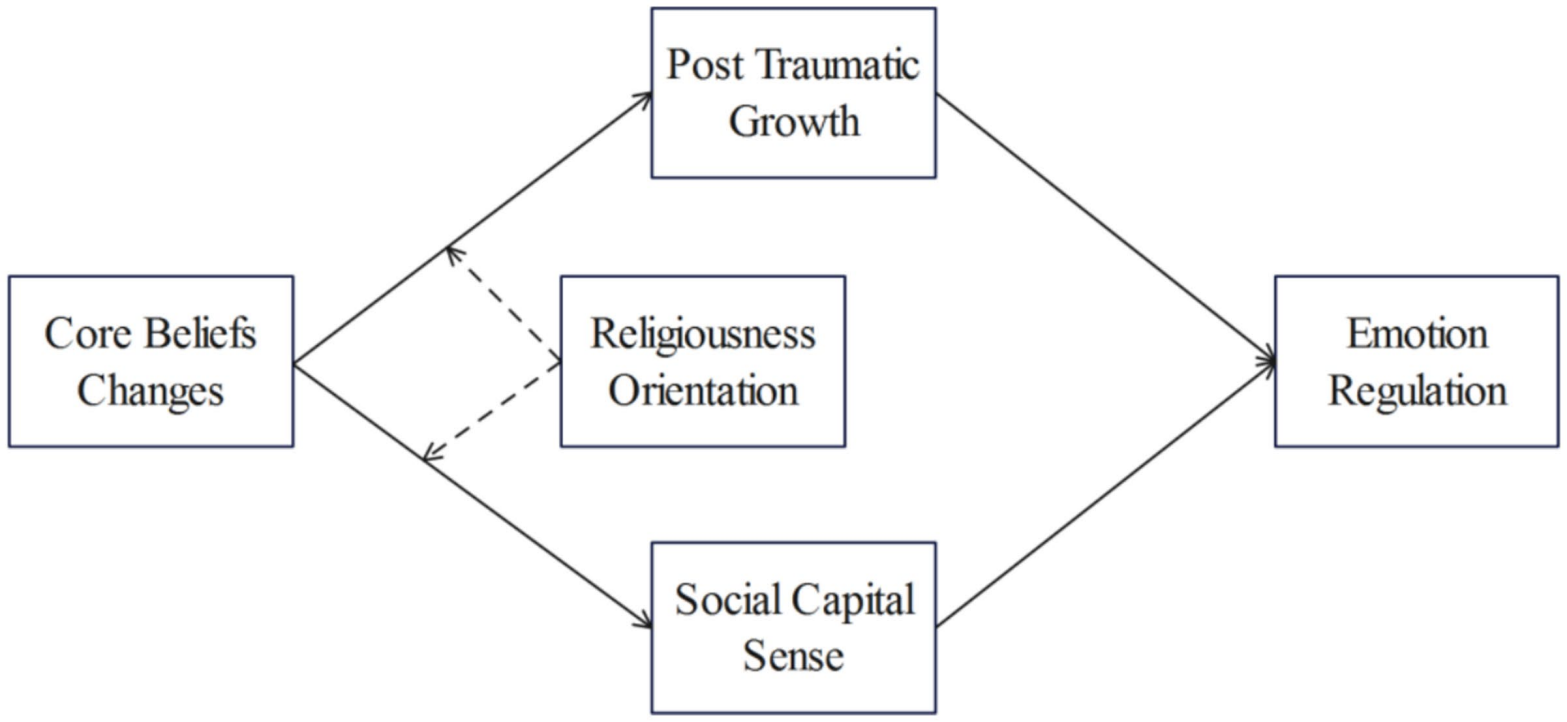
Theoretical model.

The overall theoretical hypotheses are shown in Figure 1.

## 3 Research methods

### 3.1 Research sample

This study employed a questionnaire survey method, collecting data through a combination of targeted sampling and snowball sampling. The data were obtained from residents of Shigatse City, Tibet, China, which experienced an earthquake on January 7, 2025. Respondents included both religious and non-religious individuals. Questionnaires were distributed to religious respondents through local religious associations and religious WeChat groups. The remaining questionnaires were distributed through disaster relief WeChat groups, community WeChat groups, and offline channels. Prior to questionnaire distribution, respondents were informed of the purpose of this survey. All respondents were anonymized throughout the survey process. Before participating in the questionnaire survey, respondents were informed that they needed to confirm they had been significantly affected by this earthquake.

To avoid potential common method bias, data collection employed a time-lagged approach divided into two stages. The first stage was conducted from August 4, 2025, to August 11, 2025, 6 months after the earthquake occurred. Respondents’ demographic information, core beliefs changes questionnaire, and religiousness orientation questionnaire were collected. A total of 629 valid questionnaires were obtained in the first stage, with an effective response rate of 87%. The second stage collected respondents’ post traumatic growth questionnaire, social capital sense questionnaire, and emotion regulation questionnaire. The two stages were separated by 2 weeks. The 14-day interval was implemented to reduce common method variance and does not establish temporal precedence between variables or enable causal inference. To ensure precise matching of data from the two time points, the variable data obtained from the second survey were paired one-to-one with the respondent codes from the first survey according to coding rules, and questionnaire pairs that could not be matched were discarded. After eliminating unmatched and incomplete questionnaires, a final sample of 553 valid questionnaire pairs was obtained, with a final effective response rate of 76.5%.

### 3.2 Variable measurement

The scales used in this study are all well-established scales with high citation rates. All questionnaires were processed using the “translation-back translation” procedure to ensure the accuracy and comprehensibility of the content. All questionnaires employed a 7-point Likert scale.

Core beliefs changes were measured using the *Core Beliefs Inventory* (Cann et al., 2010), which consists of 9 items assessing respondents’ degree of belief change in nine areas: fairness, control abilities, ways of thinking and behaving, interpersonal relationships, personal strengths and weaknesses, future expectations, meaning of life, religion or spirituality, and self-identity. The items of this scale do not distinguish between positive and negative changes in core beliefs, allowing assessment of both positive and potential negative changes in respondents’ core beliefs, making it suitable for this study’s context. In this study, since religiousness orientation was examined as a moderator of the correlation between core beliefs changes and post traumatic growth, as well as the correlation between core beliefs changes and social capital sense, to avoid redundant measurement of conceptually overlapping content, item 8 (“Because of the event, I seriously examined my spiritual or religious beliefs”) was removed. The Cronbach’s *α* value for the scale consisting of the remaining eight items in this study was 0.903.

Post traumatic growth was measured using *the 21-item Posttraumatic Growth Inventory* (Tedeschi and Calhoun, 1996). This scale consists of five dimensions: Relating to Others, New Possibilities, Personal Strength, Spiritual Change, and Appreciation of Life. The 21 items of the scale are applicable to the post-disaster psychological assessment context of this study, and the Cronbach’s *α* value in this study was 0.945.

Social capital sense was measured using *the Personal Social Capital Scale* (Chen et al., 2009). This scale consists of 10 items, among which item 1 assesses respondents’ number of family members, friends, neighbors, colleagues, townsfolk, and classmates. Item 6 assesses the number of various associations and organizations in respondents’ communities. These two items are objective scoring items whose scores would not change due to changes in respondents’ core beliefs; therefore, these two items were deleted. The remaining 8 items, respectively, assess respondents’ number of people with whom they maintain regular contact, number of people they trust, number of people who can provide them with help, resources possessed by members of their social capital networks, level of community organization participation, degree of connection between community organizations and community members’ rights and interests, level of support from community organizations, and resources possessed by community organizations. After deleting the two items, the remaining 8 items adequately reflect respondents’ dynamic evaluations and feelings about the social capital they possess after the disaster. In this study, the Cronbach’s α value for the 8-item scale was 0.815.

Emotion regulation was measured using *the Emotion Regulation Questionnaire* (Gross and John, 2003). This scale consists of two dimensions, cognitive reappraisal and expressive suppression, with a total of 10 items. The Cronbach’s α value in this study was 0.847.

Religiousness orientation was measured using *the Duke University Religion Index* (Koenig and Büssing, 2010). This scale consists of 5 items that provide a quantitative assessment of respondents’ religiousness. It does not qualitatively assess whether one has religious beliefs and is applicable to both religious and non-religious individuals. The Cronbach’s α value for this scale in this study was 0.909.

Control variables included gender (0 representing female, 1 representing male), age, and education level. Education level was scored on a 5-point scale, with 1 representing below high school, 2 representing high school, 3 representing junior college, 4 representing undergraduate, and 5 representing master’s degree and doctoral degree.

## 4 Data analysis

### 4.1 Common method bias test

Harman’s single-factor test was used to examine common method bias. The results showed that the variance explained by the first factor was 21.092%, which is less than 40%. Additionally, after adding a method factor to the original factor structure, common method bias was tested again, and the results indicated that the model fit indices did not significantly improve after adding the method factor. This suggests that there is no severe common method bias in this study.

### 4.2 Confirmatory factor analysis

Confirmatory factor analysis was conducted to examine the discriminant validity of the variables. The test results showed that the five-factor model had the best fit (*χ*^2^/df = 1.096, RMSEA = 0.012, GFI = 0.915, NFI = 0.924, AGFI = 0.909). This indicates that the five variables in this study have good discriminant validity.

### 4.3 Descriptive statistical analysis

Descriptive statistical results are presented in Table 1. As shown in Table 2, core beliefs changes were significantly correlated with post traumatic growth (*r* = 0.253, *p* < 0.01). Core beliefs changes were significantly negatively correlated with social capital sense (*r* = −0.278, *p* < 0.01). Post traumatic growth was significantly correlated with emotion regulation (*r* = 0.227, *p* < 0.01). Social capital sense was significantly correlated with emotion regulation (*r* = 0.146, *p* < 0.01). The correlation between core beliefs changes and emotion regulation was not significant (*r* = 0.019, *p* > 0.05). The hypotheses proposed in this study were preliminarily verified.

**Table 1:** Confirmatory factor analysis results.

Models	*χ*^2^/df	RMSEA	GFI	NFI	AGFI
Five-factor model	1.096	0.012	0.915	0.924	0.909
Four-factor model	2.358	0.045	0.801	0.833	0.784
Three-factor model	5.032	0.079	0.526	0.645	0.486
Two-factor model	6.279	0.095	0.488	0.556	0.451
Single-factor model	9.015	0.116	0.371	0.351	0.318

Five-factor model: CBC, PTG, SCS, RO, ER; four-factor model: CBC+RO, PTG, SCS, ER; three-factor model: CBC + PTG + SCS, RO, ER; two-factor model: CBC + RO + PTG + SCS, ER; single-factor model: CBC + RO + PTG + SCS + ER.

**Table 2:** Descriptive statistics and correlation analysis results.

Variables	Mean	SD	Gender	Age	Education level	Core beliefs changes	Post traumatic growth	Social capital sense	Religiousness orientation
Gender	0.494	0.5							
Age	36.062	7.72	0.027						
Education level	2.826	0.942	0.032	−0.11**					
Core beliefs changes	2.702	0.743	−0.019	−0.041	−0.003				
Post traumatic growth	2.853	0.74	−0.011	0.019	0.024	0.253**			
Social capital sense	4.401	1.106	−0.02	0.014	0.062	−0.278**	−0.102**		
Religiousness orientation	2.575	1.213	0.013	0.028	−0.048	−0.018	0.011	0.052	
Emotion regulation	4.29	0.976	−0.088*	−0.023	0.035	0.019	0.227**	0.146**	−0.027

*p < 0.05, **p < 0.01.

### 4.4 Hypothesis testing

Hypotheses 1–4 were tested using Mplus 8.3 software. As shown in Table 3, in Model 1, core beliefs changes were significantly positively correlated with post traumatic growth (*B* = 0.243, SE = 0.042, *p* < 0.001). In Model 4, post traumatic growth was significantly positively correlated with emotion regulation (*B* = 0.225, SE = 0.051, *p* < 0.001). Therefore, Hypothesis 1 and Hypothesis 3 were supported.

**Table 3:** Regression analysis results for hypotheses 1–4.

Variable	Post traumatic growth	Social capital sense	Emotion regulation		
	Model 1	Model 2	Model 3	Model 4	Model 5
Gender	−0.006	−0.029	−0.108*	−0.092*	−0.102*
Age	0.03	0.015	−0.014	−0.026	−0.02
Education level	0.027	0.065	0.055	0.042	0.031
Core beliefs changes	0.243***	−0.278***	0.015		
Post traumatic growth				0.225***	
Social capital sense					0.151***
*R*^2^	0.065	0.085	0.009	0.051	0.029
*F*	9.259***	12.016***	1.356	6.252***	3.019**

*p < 0.05, **p < 0.01, ***p < 0.001.

In Model 2, core beliefs changes were significantly negatively correlated with social capital sense (*B* = −0.278, SE = 0.057, *p* < 0.001). In Model 5, social capital sense was significantly positively correlated with emotion regulation (*B* = 0.151, SE = 0.035, *p* < 0.001). Therefore, Hypothesis 2, Hypothesis 4 were supported.

The low *R*^2^ values in the model indicate the low explanatory power of the predictors and the possible existence of other influencing factors not discussed in this study. Although the above tests suggest that the hypotheses were supported, the correlations should not be simply interpreted as causal relationships (Rubin, 1974; Bailey et al., 2024). Furthermore, although the above test results theoretically suggest that post traumatic growth and social capital sense may mediate the relationship between core beliefs changes and emotion regulation, the research method and cross-sectional data cannot support potential mediation effects. Therefore, further testing of the relevant mediation effects in this study would be meaningless.

As shown in Table 4, the interaction term between core beliefs changes and religiousness orientation was significantly correlated with post traumatic growth (*B* = 0.151, *p* < 0.001). The interaction term between core beliefs changes and religiousness orientation was significantly correlated with social capital sense (*B* = 0.146, *p* < 0.001). Therefore, Hypotheses 5 and 6 were supported.

**Table 4:** Regression analysis results for hypotheses 5 and 6.

Variable	Post traumatic growth	Social capital sense
Gender	−0.005	0.031
Age	0.007	0.023
Education level	0.003	0.006
Core beliefs changes	0.232***	−0.303***
Religiousness orientation	0.02	0.053
Core beliefs changes × religiousness orientation	0.151***	0.146***
*R*^2^	0.092	0.11
*F*	10.312***	11.219***

***p < 0.001.

**Figure 2:**
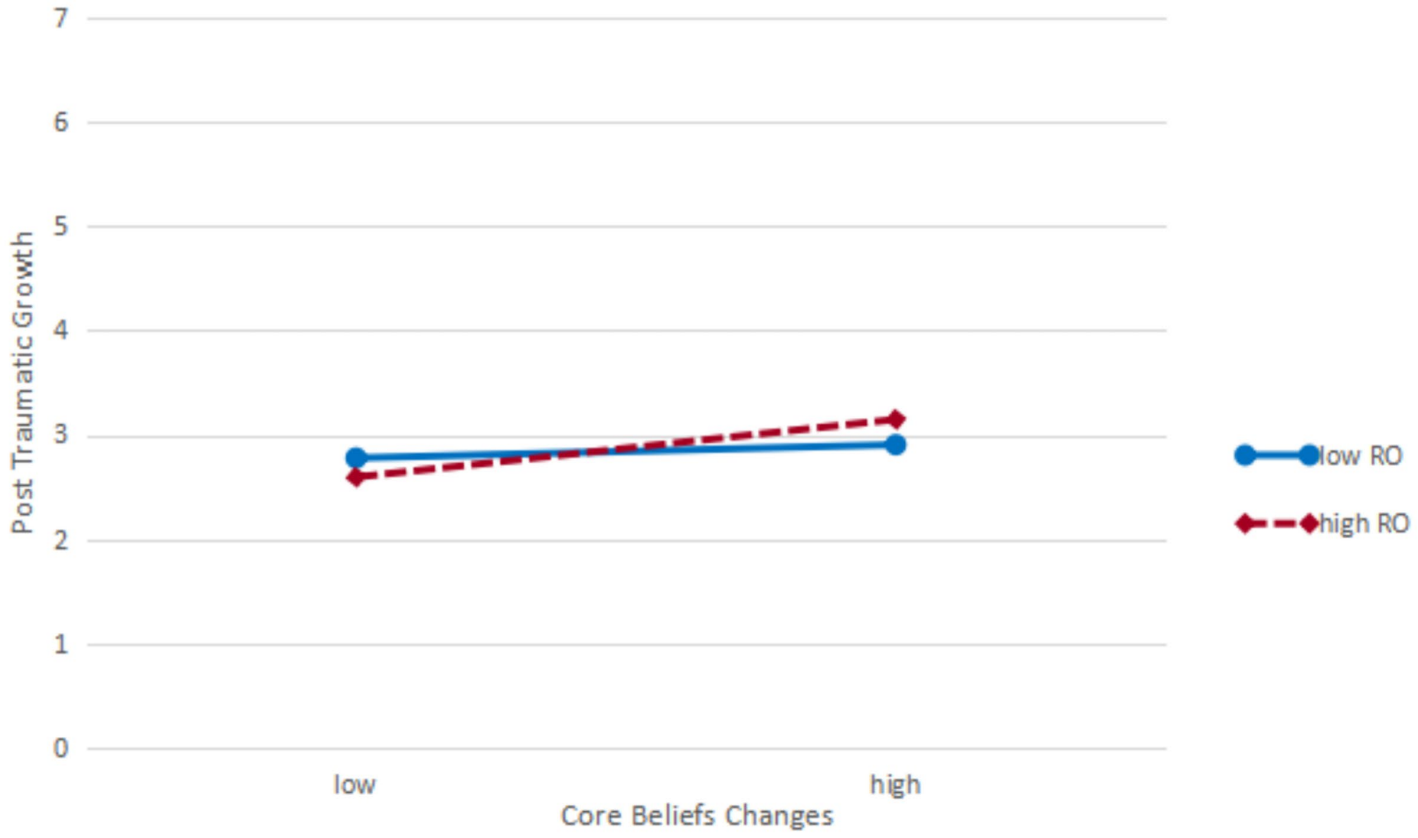
Simple slope plot of the moderating effect of religiousness orientation on the relationship between core beliefs changes and post traumatic growth.

**Figure 3:**
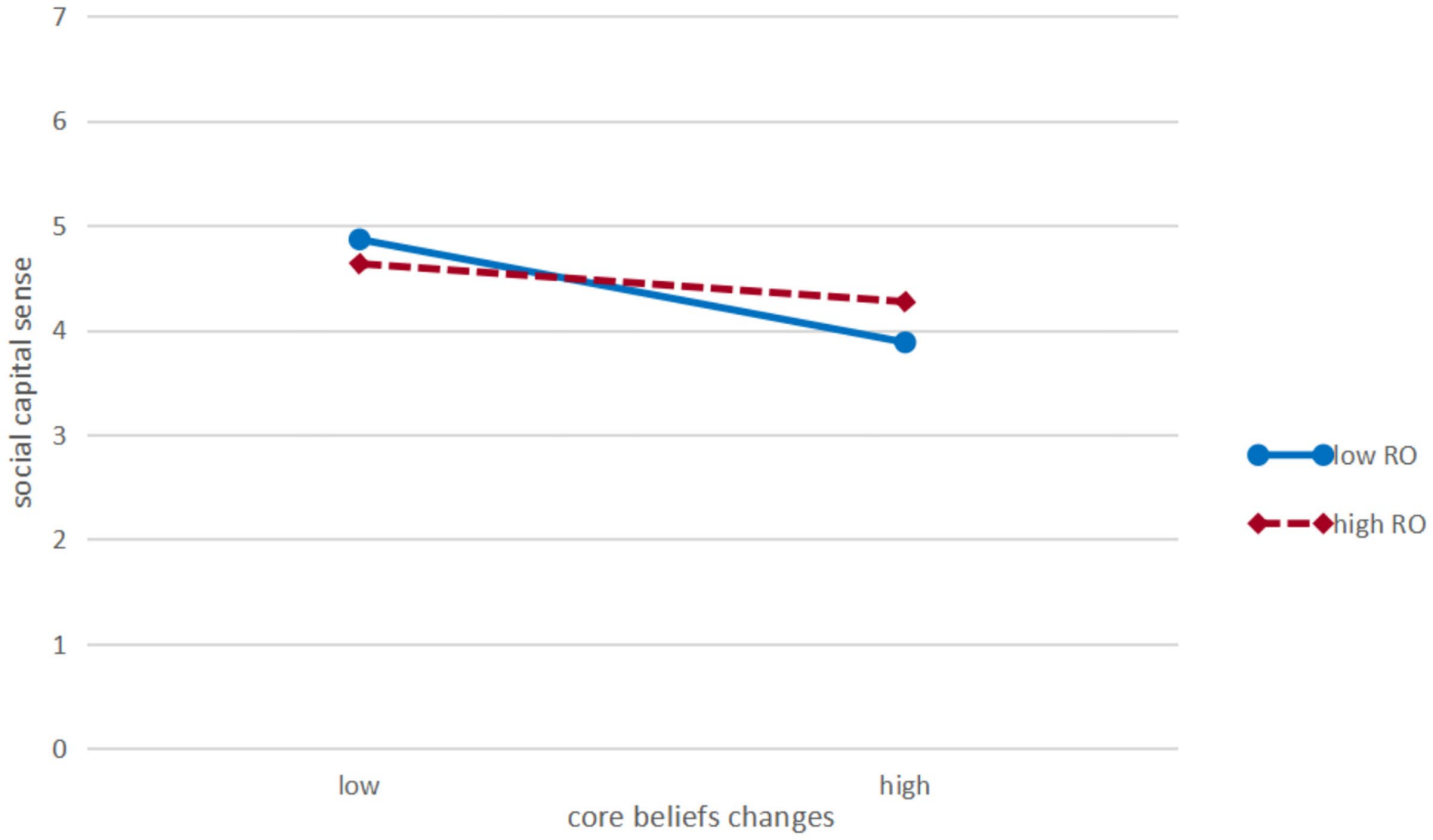
Simple slope plot of the moderating effect of religiousness orientation on the relationship between core beliefs changes and social capital sense.

Moderation plots were constructed at one standard deviation above and one standard deviation below the mean (see Figures 2, 3). As shown in Figures 2, 3, under the condition of high religiousness orientation, the correlation between core beliefs changes and post traumatic growth was stronger, and the negative correlation with social capital sense was weaker.

### 4.5 Sub-dimension correlations

Further analysis was conducted on the correlations among the sub-dimensions of the factors. Based on the sub-dimension divisions of the scales used in this study, a total of nine sub-dimensions were identified: CBC (single dimension), PTG (relating to others), PTG (new possibilities), PTG (personal strength), PTG (spiritual change), PTG (appreciation of life), SCS (single dimension), ER (cognitive reappraisal), and ER (expressive suppression).

From Table 5, it can be observed that core beliefs changes were significantly correlated with all dimensions of post traumatic growth, further confirming the hypotheses in Section 2.2. Cann et al. (2010) demonstrated, from both qualitative and quantitative perspectives, the close correlation and strict distinction between the *Core Beliefs Inventory* (Cann et al., 2010) and the *21-item Posttraumatic Growth Inventory* (Tedeschi and Calhoun, 1996) used in this study.

**Table 5:** Sub-dimension correlation analysis.

Sub-dimensions	Mean	SD	CBC	PTG (RO)	PTG (NP)	PTG (PS)	PTG (SC)	PTG (AL)	SCS	ER (CR)
CBC	2.706	0.743								
PTG (RO)	2.721	1.06	0.265**							
PTG (NP)	2.6	0.581	0.221**	0.84**						
PTG (PS)	3.25	0.908	0.262**	0.82**	0.76**					
PTG (SC)	2.5	0.702	0.259**	0.794**	0.745**	0.726**				
PTG (AL)	3.429	0.53	0.199**	0.849**	0.801**	0.796**	0.729**			
SCS	4.401	1.106	−0.29**	−0.165**	−0.078	−0.14**	−0.058	−0.059		
ER (CR)	4.245	1.089	0.115**	0.359**	0.233**	0.375**	0.309**	0.286**	−0.155**	
ER (ES)	4.358	0.802	−0.094*	−0.068	−0.073	−0.065	−0.063	−0.08	0.327**	−0.235**

*p < 0.05, **p < 0.01.

The negative correlation between post traumatic growth and social capital sense primarily originated from two dimensions: relating to others and personal strength.

All dimensions of post traumatic growth were significantly positively correlated with cognitive reappraisal, but none were significantly correlated with expressive suppression. Social capital sense was significantly negatively correlated with cognitive reappraisal and significantly positively correlated with expressive suppression, confirming the negative correlation between social capital sense and cognitive reappraisal proposed in Section 2.3.

The two dimensions of emotion regulation in this study’s sample were negatively correlated. Gross and John (2003) found no significant correlation between these two dimensions in their sample of American university students. They suggested that cognitive reappraisal and expressive suppression are two independent dimensions in daily life. The findings of this study reflect the influence of cultural differences in the sample and specific triggering events on the selection of emotion regulation strategies.

Additionally, further analysis revealed that gender (male = 1) was negatively correlated with ER (CR) (*r* = −0.085, *p* < 0.05) and also negatively correlated with ER (ES) (*r* = −0.092, *p* < 0.05). In the post-earthquake environment, male residents in the Shigatse region of Tibet used both emotion regulation strategies relatively less frequently.

## 5 Conclusions, discussion, and suggestions

### 5.1 Conclusions and discussion

This study focuses on residents in the 2025 Shigatse earthquake-stricken areas, exploring the relationships among core beliefs changes, post traumatic growth, social capital sense, emotion regulation, and religiousness orientation. The study innovatively proposes and verifies the dynamic role of “social capital sense” in post-disaster psychological mechanisms, addressing the neglect of subjective perception in traditional social capital research. It expands the dimensions of social capital research, particularly suitable for the dynamic assessment of post-disaster psychological changes. The main conclusions are as follows:

There are two opposing relationships between core beliefs changes and emotion regulation: in Relationship 1, core beliefs changes are positively correlated with post traumatic growth, and post traumatic growth is positively correlated with emotion regulation. In Relationship 2, core beliefs changes are negatively correlated with social capital sense, and social capital sense is positively correlated with emotion regulation.

Religiousness orientation plays a significant moderating role in both relationships: For individuals with high religiousness orientation, the positive correlation between core beliefs changes and post traumatic growth is stronger, while the negative correlation between core beliefs changes and social capital sense is weaker.

The research results demonstrated the relationships among core beliefs changes, post traumatic growth, and cognitive reappraisal. In post-disaster environments, affected residents may simultaneously experience these three psychological motivations. However, although these three psychological factors are closely related, they maintain strict distinctions (Cann et al., 2010).

This study found that in a post-disaster environment, post traumatic growth and social capital sense are independently associated with the two dimensions of emotion regulation:

The decrease in social capital sense induced by post-disaster helplessness is significantly correlated with cognitive reappraisal. This aligns with previous research suggesting that “individuals in Asian cultures suppress emotions to maintain interpersonal relationships” (Butler and Gross, 2009; Deng et al., 2019). Conversely, a decline in post-disaster social capital sense may lead to a reduction in interpersonal relationship maintenance. The correlation between social capital sense and cognitive reappraisal is not significant. Expressive suppression among East Asian individuals is closely linked to collectivist cultural norms. Influential factors include not only the maintenance of interpersonal harmony, but also the preservation of one’s image within the group (termed “face” in the East Asian context) (Ho, 1976), and hierarchical obedience within vertical collectivism (Matsumoto et al., 2008). However, these studies were either qualitative in nature or conducted under non-special circumstances using everyday-life samples, and they all treated the relationship between social capital (or interpersonal relationships) and cognitive reappraisal as a static or trait-based concept. This study found that, under the unique triggering circumstances of a disaster, although social capital sense among post-disaster residents in Tibet remained significantly correlated with expressive suppression, both core beliefs changes and cognitive reappraisal (uncommon factors in daily life) were significantly negatively correlated with social capital sense. This suggests that in such special environments, a decline in social capital sense may also weaken expressive suppression.

The five sub-dimensions of post traumatic growth— PTG (RO), PTG (NP), PTG (PS), PTG (SC), and PTG (AL)—are all significantly positively correlated with cognitive reappraisal (*p* < 0.01), but none show a significant correlation with expressive suppression. Theoretically, in a post-disaster context, post traumatic growth and emotion regulation (cognitive reappraisal) may have a bidirectional facilitative effect.

In the study by Gross and John (2003) on emotion regulation in everyday contexts, using a sample of American university students, the correlation between the two dimensions of emotion regulation was not significant, indicating they are two independent dimensions. Individuals who frequently use one strategy do not necessarily frequently use the other. They found that suppression reduces the expression of negative emotions but also inhibits the expression of positive emotions. Furthermore, suppression only reduces expression but does not help diminish negative emotions, which may persist and accumulate over time. Suppression requires individuals to continuously manage negative emotions, and these repeated efforts may consume cognitive resources. In this study, the negative correlation between the cognitive reappraisal sub-dimension and the Expressive Suppression sub-dimension confirms this issue. In a post-disaster environment, emotional release can help avoid depression, anxiety, and post-traumatic stress disorder (Kwon et al., 2020), suggesting that expressive suppression may not have positive effects in such contexts.

When emotion regulation is assessed using cognitive reappraisal and expressive suppression, gender differences are widespread and diverse in type. In the study by Zhang and Bian (2020) on Chinese university students, no significant gender differences were found in expressive suppression, but males scored higher than females in cognitive reappraisal. In the study by Megías-Robles et al. (2019) on a Spanish resident sample, females scored higher than males in cognitive reappraisal and lower than males in expressive suppression. In the study by Melka et al. (2011) on American university students, no significant gender differences were found in cognitive reappraisal, but females scored lower than males in expressive suppression. In all these studies, the samples were drawn from everyday life settings (or normal daily contexts). The post-disaster context of this study provides a new sample type for measuring emotion regulation (CR & ES). The results show that gender (male = 1) is negatively correlated with both cognitive reappraisal and expressive suppression. This result aligns with previous research: traditional masculine norms negatively influence motivation for psychological adaptation (Piatkowski et al., 2024), and post-disaster contexts amplify these negative effects (Mokhwelepa and Sumbane, 2025).

Although the Duke University Religion Index (Koenig and Büssing, 2010) used in this study demonstrates strong applicability and does not distinguish between religious categories, the lack of a religiousness orientation scale specifically tailored to our sample is a limitation. Furthermore, the snowball sampling method, which recruited participants through religious and community organizations, limits the generalizability of the results to individuals with lower levels of social and religious embeddedness. During the survey process, it was identified that some residents in remote areas were inaccessible or did not use communication devices; thus, the study may have overlooked certain minority groups. Future research should consider adopting longitudinal designs and mixed methods to better capture the complexity of psychological adaptation.

### 5.2 Recommendations

In past large-scale natural disasters, religious organizations have actively participated in post-disaster psychological assistance and community reconstruction, playing an irreplaceable role (Okyere et al., 2024; Scheffert and Ellor, 2022).

It is recommended that religious organizations provide victims with a transcendent framework of meaning (e.g., “disasters are a test,” “life has a higher meaning”), helping individuals integrate their traumatic experiences into their life narratives and promote the positive reconstruction of core beliefs. Through activities such as prayer meetings and collective meditation, religious organizations can offer victims a space for emotional catharsis and reflective rumination, facilitating the transition from intrusive to reflective rumination, helping victims deal with crises of faith and existential issues, and enhancing their sense of hope and meaning. Religious organizations often have the capacity for cross-regional and cross-class connections; it is recommended to leverage this to promote the flow of resources and information exchange, helping victims access broader social support. Furthermore, by utilizing the credibility and organizational capacity of religious institutions, they can assist governments and NGOs in rescue efforts, enhancing victims’ trust in the aid system.

In post-disaster environments, interpersonal social capital is not an absolute positive. Although short-term expressive suppression can avoid interpersonal conflicts or social sanctions caused by emotional expression, it also increases emotional distance from others. The expressive suppression required to maintain interpersonal social capital may exacerbate the accumulation of negative emotions and lead to adverse outcomes (Gross and John, 2003), particularly in Asian contexts. Therefore, it is recommended to provide timely emotional counseling channels for disaster victims. At the same time, the potential bidirectional reinforcing effect between post traumatic growth and emotion regulation (cognitive reappraisal) should be fully utilized.

For individuals with a high religious orientation, their faith resources can be leveraged to promote post taumatic growth; for those with a low religiousness orientation, the construction and maintenance of social support systems should be strengthened. Post-disaster psychological assistance should focus on enhancing individuals’ perception of social support, for example, by rebuilding trust and a sense of belonging through community activities and group counseling. The study found that males have weaker post-disaster emotion regulation abilities; it is recommended to develop emotional expression and regulation training targeted at men to prevent psychological problems caused by emotional suppression. Post-disaster psychological reconstruction is a long-term process; it is recommended to establish a long-term mechanism to regularly assess residents’ psychological states and provide ongoing support.

## Data Availability

The data presented in this study are not publicly available. Requests for access to the data may be directed to the corresponding author.
